# Simultaneous Detection of CNVs and SNVs Improves the Diagnostic Yield of Fetuses with Ultrasound Anomalies and Normal Karyotypes

**DOI:** 10.3390/genes11121397

**Published:** 2020-11-25

**Authors:** Qingwei Qi, Yulin Jiang, Xiya Zhou, Hua Meng, Na Hao, Jiazhen Chang, Junjie Bai, Chunli Wang, Mingming Wang, Jiangshan Guo, Yunshu Ouyang, Zhonghui Xu, Mengsu Xiao, Victor Wei Zhang, Juntao Liu

**Affiliations:** 1 Department of Obstetrics and Gynecology, Peking Union Medical College Hospital, Peking Union Medical College & Chinese Academy of Medical Sciences, Beijing 100730, China; yulinj@gmail.com (Y.J.); zhouxiya@pumch.cn (X.Z.); hnqxd@sina.com (N.H.); 13901365269@163.com (J.L.); 2 Department of Ultrasound, Peking Union Medical College Hospital, Peking Union Medical College & Chinese Academy of Medical Sciences, Beijing 100730, China; menghua_pumch@163.com (H.M.); oy81@163.com (Y.O.); xzhpumch@hotmail.com (Z.X.); xiaomengsu_2000@sina.com (M.X.); 3 Department of Medical Research Center, Peking Union Medical College Hospital, Peking Union Medical College & Chinese Academy of Medical Sciences, Beijing 100730, China; changjiazhen@outlook.com; 4 Be Creative Lab Co., Ltd. Beijing 101111, China; benny_2009@163.com (J.B.); aolai1211@163.com (M.W.); 13717568611@163.com (J.G.); 5 AmCare Genomics Lab, Guangzhou 510335, China; wangchli1987@163.com (C.W.); Victor.w.zhang@amcarelab.com.cn (V.W.Z.)

**Keywords:** fetal ultrasound anomalies, prenatal diagnosis, CMA, CNV-seq, clinical exome sequencing (CES)

## Abstract

The routine assessment to determine the genetic etiology for fetal ultrasound anomalies follows a sequential approach, which usually takes about 6–8 weeks turnaround time (TAT). We evaluated the clinical utility of simultaneous detection of copy number variations (CNVs) and single nucleotide variants (SNVs)/small insertion-deletions (indels) in fetuses with a normal karyotype with ultrasound anomalies. We performed CNV detection by chromosomal microarray analysis (CMA) or low pass CNV-sequencing (CNV-seq), and in parallel SNVs/indels detection by trio-based clinical exome sequencing (CES) or whole exome sequencing (WES). Eight-three singleton pregnancies with a normal fetal karyotype were enrolled in this prospective observational study. Pathogenic or likely pathogenic variations were identified in 30 cases (CNVs in 3 cases, SNVs/indels in 27 cases), indicating an overall molecular diagnostic rate of 36.1% (30/83). Two cases had both a CNV of uncertain significance (VOUS) and likely pathogenic SNV, and one case carried both a VOUS CNV and an SNV. We demonstrated that simultaneous analysis of CNVs and SNVs/indels can improve the diagnostic yield of prenatal diagnosis with shortened reporting time, namely, 2–3 weeks. Due to the relatively long TAT for sequential procedure for prenatal genetic diagnosis, as well as recent sequencing technology advancements, it is clinically necessary to consider the simultaneous evaluation of CNVs and SNVs/indels to enhance the diagnostic yield and timely TAT, especially for cases in the late second trimester or third trimester.

## 1. Introduction 

Fetal structural abnormalities detected by ultrasound can be identified in 2% to 3% of pregnancies. A significant portion of these fetuses have an underlying genetic etiology associated with a spectrum of mutation types, including chromosome aneuploidy, copy number variations (CNVs), uniparental disomy (UPD) and single nucleotide variation (SNV)/small insertion-deletions (indels). In general, aneuploidies are found in 8% to 10% of unselected fetuses with abnormal ultrasound results, while microdeletions/microduplications are identified in another 6% by chromosomal microarray analysis (CMA) [1]. If negative, further testing by exome sequencing (ES), either whole exome sequencing (WES) or clinical exome sequencing (CES), can provide a positive detective rate in 8.5% to 33% of fetal ultrasound anomalies cases [2,3,4,5,6]. Thus, the cumulative diagnostic yield is estimated to be approximately in the range of 22.5% to 49% for cases with a suspected genetic etiology containing these three major disease-causing mutation types. 

Currently, the routine procedure for prenatal genetic diagnosis of cases with fetal anomalies is a sequential procedure in most of prenatal clinics [7], namely, performing karyotyping to reveal the presence or absence of aneuploidy and higher resolution CMA to detect CNVs in fetuses with a normal karyotype. When these results were uninformative, ES was performed to detect exonic SNVs/indels. However, this step-wise procedure has a relatively long turnaround time (TAT, 4–8 weeks). More importantly, the prenatal genetic workup is incomplete when analysis only included aneuploidies and CNVs. Without exonic SNVs/indels, a genetic diagnosis may not be reached that may affect management such as genetic counseling. 

With rapid adoption of new technologies in clinical settings, for the identification of microdeletions/microduplications genomic disorders, CNVs detection has been traditionally performed by microarray-based CMA approach. Recently, low-pass genome sequencing (CNV-seq) has been gradually applied in prenatal diagnosis because of its high throughput, high resolution, and relatively low cost [8,9,10]. As for monogenic disorders, both WES and CES are used as effective assays for detecting exonic SNVs/indels. Based on the recommended genomic medicine framework from ClinGen, clinical applications are focused on the essential genes for monogenic disease, which are associated with known diseases. CES is commonly defined as a targeted gene panel comprised of most of the known human disease-causative genes in the Online Mendelian Inheritance in Man (OMIM) database [11,12]. Most recent studies also have shown that monogenic diseases account for a large proportion of fetal structural anomalies [2,3,4,5,6], which could be recurrent in next pregnancy. 

More importantly, it is a challenging task to make a definite genetic diagnosis based on the limited fetal phenotypic features, as compared to pediatric patients. Moreover, in a prenatal setting, a short TAT and comprehensive genetic evaluation is required for timely and accurate genetic counseling, particularly when the ultrasound anomalies were detected in the late second trimester or even later. Herein, we evaluated the clinical utility of simultaneous detection of CNVs and SNVs/indels in fetuses with a normal karyotype and ultrasound anomalies. Our current procedure was comprised of the detection of CNV by CMA and/or CNV-seq, and in parallel the identification of SNVs/indels by trio-based CES and/or WES. We conducted the prospective analysis to obtain the molecular diagnosis by CMA combining with CES in a cohort of 83 fetal ultrasound anomalies.

## 2. Materials and Methods

### 2.1. Cohort

**Figure 1:**
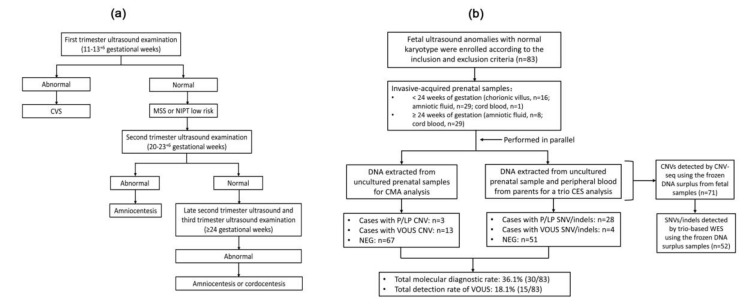
Workflow of the ultrasound examination and prenatal genetic diagnosis. (**a**) The workflow of routine ultrasound examination during pregnancy. CVS, chorionic villus sampling; MSS, maternal serum screening; NIPT, non-invasive prenatal testing. (**b**) CMA and CES were used for the simultaneous detection of CNVs and SNVs/indels. CMA, chromosomal microarray analysis; CES, clinical exome sequencing; P/LP, pathogenic/likely pathogenic; VOUS, variants of uncertain significance.

The study protocol was approved by the medical ethics committee of the Children’s Hospital affiliated to Zhejiang University. In this prospective observational study, a total of 83 fetuses were enrolled for prenatal diagnosis at the Peking Union Medical College Hospital from May 2016 to July 2019. The workflow of the ultrasound examination was as follows (Figure 1a): the first trimester ultrasound was performed between 11 to 13 + 6 gestational weeks, and the second trimester (20 to 23 + 6 weeks) was performed on pregnant women with a negative result from non-invasive prenatal testing (NIPT) or maternal serum screening (MSS). The late second trimester (24 to 28 weeks) and the third trimester (30 to 32 weeks) ultrasound examination were conducted after those tests. All cases met the following inclusion criteria: (1) informed consent procedure for prenatal genetic diagnosis from the parents was provided according to program grant for National Key R&D Program of China “assessing the national integrated birth defect prevention model” (grant number 2018YFC1002704, IRB/EC number 2018-IRB-076); (2) singleton pregnancy and a fetus with at least one ultrasonic structural anomaly; (3) fetal sample was obtained through an invasive procedure, including chorionic villus sampling (CVS), amniocentesis or cordocentesis; (4) prenatal genetic diagnosis including karyotyping, CMA and trio-based CES was performed in parallel; (5) all of the above-mentioned testing were performed on each prenatal sample successfully; and (6) karyotyping results were normal. The exclusion criteria included: (1) parents that refused to accept the procedure of genetic analysis simultaneously; and (2) abnormal karyotype results. Fetal samples were obtained by CVS from 16 pregnancies at 12 to 14 weeks of gestation, amniocentesis from 37 pregnancies at 17 to 27 weeks of gestation, and cordocentesis from 30 pregnancies at 23 to 33 weeks of gestation. All procedures were performed via ultrasonography guidance. The peripheral blood of the parents was sampled for a trio analysis. 

Fetal genomic DNA was extracted from uncultured samples for CMA and CES. DNA surplus was stored at −20 ℃. CNV-seq and trio WES were performed when sufficient genomic fetal and parents’ DNA was available. The study design was shown in Figure 1b.

### 2.2. Karyotype Analysis

Chromosome analysis using GTG-banding was completed according to standard procedures. A total of 10 metaphase cells were analyzed. Karyotype summaries were made according to the International System for Human Cytogenetic Nomenclature. 

### 2.3. Chromosomal Microarray Analysis (SNP Array)

Genomic DNA from fetuses was extracted from uncultured amniotic fluid, villus or cord blood using the QIAamp DNA Blood Mini Kit (Qiagen, Valencia, CA, USA). The DNA was digested, ligated with adaptors, amplified, purified, and labeled with biotin. Then, the DNA was hybridized to the Affymetrix^®^ CytoScanTM 750K Array (Affymetrix, Santa Clara, CA, USA). The arrays were washed with the Affymetrix GeneChip^®^ Fluidics Station 450 and scanned with an Affymetrix GeneChip^®^ Scanner 3000 according to the manufacturer’s protocol. CEL files obtained by scanning the arrays were analyzed with the Chromosome Analysis Suite (ChAS) v33.1 software. The GRCh37 (hg19, http://genome.ucsc.edu/) genome was used for annotation of CNVs. 

### 2.4. CNV-Sequencing (CNV-Seq)

Low-coverage whole genome sequencing (~0.5–1× coverage) was used to detect CNVs. Genomic DNA was extracted, followed by random fragmentation and short read sequencing using the Illumina NextSeq500 or NovaSeq6000 sequencer (Illumina, San Diego, CA, USA). Sequencing reads were cleaned by removing read when a base quality less than QC20 and mapped to the reference human genome version hg19. CNVs were evaluated by an in-house bioinformatics pipeline using read counts and Z-scores (AmCare Genomics Lab, Guangzhou, China). Briefly, the coverage profiles for each sample of window of 25 kb across human genome were generated for absolute read count. The relative coverage depth was calculated across gender matched samples first, which usually contain at least 5 males and 5 females for gender matched normalization without GC correction. Z-score were calculated for each 25 kb window for each sample across the batch. When at least four continuous 25 kb windows with Z-score is either below −2 or above 2 are marked for further manual examination. The high variations regions indicating highly homologous or repeat regions across different samples were excluded for further analysis. The interpretation of CNVs was based on the Database of Genomic Variants (DGV, http://dgv.tcag.ca/dgv/app/home), DECIPHER (https://decipher.sanger.ac.uk/), OMIM (https://www.omim.org/) and peer-reviewed literatures. The resolution was 100 kb with bin size of 25 kb.

### 2.5. Clinical Exome Sequencing 

Custom-designed NimbleGen SeqCap probes (Roche NimbleGen, Madison, WI, USA) were used for in-solution hybridization to enrich target sequences, which included coding exons for about 5000 clinically relevant disease causing genes [13,14]. The genes were selected based on reports in OMIM, HGMD, and peer-reviewed literatures. Known pathogenic variants in deep intronic and other non-coding regions in targeted genes were also included. Enriched DNA samples were indexed and sequenced on an Illumina sequencer (San Diego, CA, USA). The average coverage depth was 200× with >98% of the target regions covered by at least 20 reads. Detailed analysis information can be found from our previous publications [15,16,17]. Briefly, Data were filtered to generate “clean reads” by removing adapters and reads with base quality of less than Q20. Sequencing reads were mapped to the reference human genome version hg19. Nucleotide changes observed of aligned reads were called and reviewed by using NextGENe software (Version 2.4.1.2) (SofGenetics, State College, PA, USA). Sequence variants were annotated using population and literature databases, including GnomAD (https://gnomad.broadinstitute.org/), ClinVar (https://www.ncbi.nlm.nih.gov/clinvar/), OMIM, and others. The variants with minor allele frequency (MAF) >1% in Asian population were filtered out. The interpretation of pathogenicity of variants was evaluated according to the American College of Medical Genetics (ACMG) guidelines [18]. Sanger sequencing was used to verify the suspected mutation sites in fetus and parents. 

### 2.6. Whole Exome Sequencing

In all index cases, libraries of genomic DNA samples were prepared using the Agilent Sureselect Human All Exon v5 kit (Agilent Technologies, Santa Clara, CA, USA), and were sequenced on a HiSeq instrument (Illumina, San Diego, CA, USA) according to the manufacturer’s recommendations. Raw data were processed by NextGENe for alignment (SofGenetics, State College, PA, USA). The alignment, variant filtration and interpretation process is similar to these of Clinical exome sequencing. The average coverage depth was about 80–100×. Sequence variants were annotated using population and literature databases, including GnomAD, Clinvar, OMIM, and others. Classification of variants was performed with reference to the guideline recommended by the ACMG [18]. Sanger sequencing was used to verify the suspected mutation sites in fetus and parents.

## 3. Results 

### 3.1. Cohort Characteristics 

Eighty-three singleton pregnancies with fetal ultrasound anomalies were included in this study. These fetuses were assessed at a median gestational age of 22 weeks (range 12–33). The fetuses were categorized into 11 phenotypic groups based on the abnormalities in different organ systems detected by ultrasound. These ranged from isolated anomaly (*n* = 61) to multi-system anomalies in which two or more systematic anomalies were detected (*n* = 22). The case number of each category was as following: facial anomaly (*n* = 4), brain anomaly (*n* = 13), skeletal anomaly (*n* = 13), cardiac anomaly (*n* = 4), renal anomaly (*n* = 4), cystic hygroma (*n* = 4), increased nuchal translucency (NT ≥ 3 mm, *n* = 5), hydrops (*n* = 8), early-onset fetal growth restriction (FGR, *n* = 5), fetal overgrowth (*n* = 1), and multi-systemic anomalies (*n* = 22) (Table 1). Prenatal sample types in this study included chorionic villus (*n* = 16), amniotic fluid (*n* = 37), and umbilical cord blood (*n* = 30).

### 3.2. Diagnostic Yield by Simultaneous Detections of CNVs and SNV/Indels Mutations

Pathogenic CNVs were identified in 3 fetuses and variants of uncertain significance (VOUS) detected in 13 fetuses. The mode of inheritance was confirmed by further CMA parental testing in 9 of the 16 families, while the other parents refused further testing. The 3 pathogenic CNVs were characterized as a *de novo* deletion at 16p11.2 in an increased NT group (case #12), a paternally inherited 16p11.2 duplication (case #62) and a *de novo* 6q26q27 deletion in the brain anomaly group (case #76) (Table 2). Thirteen cases with VOUS CNVs were listed in Table 2.

Samples from the 83 families (parent-fetus trio) were subjected to CES for a trio analysis. Pathogenic/likely pathogenic variants relating to fetal phenotypes were identified in 27 fetuses (Table 2). Among these positive cases, validated variants in 16 fetuses were *de novo* variants in coding or splicing sequences, including 9 missense, 1 frame-shift, 4 nonsense and 2 splice-site variants. The remaining 11 fetuses had inherited variants, including one case with a homozygous variant and 8 cases with compound heterozygous variants in accordance with autosomal recessive pattern of inheritance, and 2 cases of heterozygous variants in an autosomal dominant pattern of inheritance. Moreover, SNVs/indels associated with the fetal features were identified in 5 cases and classified as VOUS (Table 2).

Taken together, pathogenic/likely pathogenic variants were identified in 30 cases (CNVs in 3 cases, SNVs/indels in 27 cases), indicating an overall molecular diagnostic rate of 36.1% (30/83) (Table 1). A comparison of the molecular diagnosis rate for subgroups was performed, yielding 69.2% (9/13) in the skeletal anomaly group, 36.4% (8/22) in the multi-systemic anomalies group, 30.8% (4/13) in the brain anomaly group, and 25.0% (2/8) in the hydrops group (Table 1). In addition, molecular diagnostic rate in the three different sample types was not significantly different, 37.5% (6/16) in CVS, 37.8% (14/37) in amniotic fluid and 33.3% (10/30) in cord blood. Moreover, two cases had both a CNV of uncertain significance and a likely pathogenic SNV (case #14 and case #15), and one case had both a VOUS CNV and SNV (case #25). It was worth noting that CNVs in seven cases with unknown genetic sources were all further analyzed and confirmed by CES data from their parental blood samples. 

### 3.3. Turnaround Time (TAT), Pregnancy Outcomes

The mean TAT of karyotyping, CMA and trio-based CES analysis was 14 days, 14 days and 14–28 days, respectively. Routine sequential testing usually takes about 6–8 weeks. Most of the testing results were sent to parents within 3–4 weeks in the present study. If the procedure from sampling to reporting is well coordinated, the overall TAT for parallel testing can be shortened to 2–3 weeks in some of our cases.

After genetic counseling, the parents elected the termination of pregnancy (TOP) in 70 cases, continued pregnancy in 10 cases until birth, and lost to follow-up in 3 cases. A total of 12 cases were given postmortem autopsy and the results of postmortem autopsy were consistent with the results of the ultrasound examination. The pregnancy outcomes in the sub-groups were shown in Table 1, and 30 cases with P/LP variants and 15 cases with VOUS were described in detail in Table 2. In case #8, the fetus with irregular shaped occipital skull and suspected meningeal encephalocele detected by the prenatal ultrasound had compound heterozygous variants (c.1421dupA; p.N474Kfs*12 and c.1421delA; p.N474Mfs*7) in the *RPGRIP1L* (NM_015272) gene. The two unreported inherited changes were frameshift variants with a premature termination codon. However, the mother undergone a pregnancy termination due to fetal exencephaly two years ago. As a result, the fetus was prenatally diagnosed to have Meckel syndrome (MIM #611561) based on the ultrasound and genetic analysis. The parents elected to terminate the current pregnancy. The autopsy showed cleft lip and palate, absence of partial skull, six fingers/toes, and bilateral polycystic kidney, which confirmed the clinical diagnosis of Meckel syndrome. 

Worth noting, in two fetuses with cystic hygroma (case #10 and case #21), the results of the karyotype, CMA and CES were all negative. The parents elected to continue the pregnancy after genetic counseling. After delivery of the two fetuses, they were clinically healthy newborn infants, and thickening of the subcutaneous neck tissue in case #10 disappeared two months.

### 3.4. CNVs Analysis by CNV-Seq Versus CMA

The detection of CNVs by CMA and CNV-seq was also compared in this study. CNV-seq was successfully performed on 71 cases with sufficient DNA. Among 71 cases, CNVs were detected in 12 cases by CNV-seq, which were coincided with CMA results (Table 3). The breakpoints of CNVs detected using CNV-seq were similar to the breakpoints detected by CMA. Sizes of these CNVs detected by CNV-seq ranged from 624 to 9791 kb, which was slightly larger (101.0% on average) than that of CMA platform (ranged from 585 to 9791 kb). The amount of DNA used in CNV-seq and CMA was approximately 20–50 ng and 50–100 ng, respectively. The cost per sample of CNV-seq (USD 300–375) was less than CMA (USD 600–750). In addition, the average TAT of CNV-seq in our study was 10 days, which was almost the same as that of CMA. 

### 3.5. SNVs/Indels Analysis by CES Versus WES

In order to compare the efficacy of analyzing monogenic disorders between CES and WES, WES was performed on 52 cases with sufficient DNA after CNV-seq testing. WES identified (likely) pathogenic variants in 15 cases and VOUS in 4 cases, which were the same as CES. None of known pathogenic variants in deep intronic regions were detected in this study.

## 4. Discussion

This study presents a cohort of fetuses with ultrasound anomalies that underwent concurrent comprehensive genomic analysis including karyotyping, CMA and CES. A total of 83 singleton pregnancies with normal fetal karyotype were enrolled in this study. Pathogenic CNVs were identified in 3 fetuses and VOUS in 13 fetuses. Pathogenic or likely pathogenic SNVs/indels variants associated with fetal features were identified in 27 fetuses while VOUS were detected in 5 fetuses. Taken together, the simultaneous analysis of CNVs and SNVs/indels yielded an overall molecular diagnostic rate of 36.1% (30/83), with an overall VOUS detection rate at 18.1% (15/83). The relatively high molecular diagnosis rate in subgroups was 69.2% (9/13) in the skeletal anomaly group, followed by 36.4% (8/22) in the multi-systemic anomaly group, and 30.8% (4/13) in the brain anomaly group. 

### 4.1. The Need of Simultaneous Detection of CNVs and SNVs/Indels for Prenatal Diagnosis

Currently, a fetus with ultrasound anomalies is routinely recommended for evaluation of genetic disorders, including chromosome aneuploidies, microdeletion/microduplication syndromes and monogenic diseases. The sequential procedures for prenatal genetic diagnosis have been adapted in many clinics. The detection of each type of genetic mutation type is accompanied with technological advancements in genomic medicine, from hypotonic solution usage for human chromosomes analysis in 1955, CMA for microdeletion/microduplication genomic disorders in 2003, and exome sequencing for monogenic diseases in 2012. The sequential events make the base of currently step-wise testing and also reflect our understanding of the pathogenesis of genetic disorders starting from low resolution at chromosome level analysis to high resolution base-pair level analysis. Thus, such technologies have been implemented in most national wide healthcare insurance systems and were recommended by medical professional guideline. However, this sequential testing generated a number of issues in clinical practice, especially in a prenatal setting. 

A relatively complete understanding of the genome has been achieved over time through the application of sequencing technologies. For given phenotype, either one or more of the three major genetic mutation types may explain the genetic cause of such disorder. Moreover, with the wide use of NIPT, chromosome aneuploidies have been detected before the appearance of ultrasound abnormalities. Chromosomal aneuploidies were less detected in practice in fetuses with ultrasound anomalies. Meanwhile, recent studies showed the monogenic causes made up to 33% of the genetic etiology in fetal ultrasound anomalies, with half of them being *de novo* SNVs/indels [2,3,4,5,6]. Microdeletions/microduplications were identified in about 6% cases (CMA) [1]. From the clinical and testing efficiency point of view, a higher yield may be achieved if the proper testing sequence is selected, but it is challenging in many specific cases. Unfortunately, in cases when CMA/CNV-seq is performed with actionable findings, the genetic workup would be typically stopped and other genetic variants missed. According to the pediatric genomic findings, a double diagnosis, even triple diagnosis has been revealed in about 5–10% of the cases. An incomplete genetic workup may apply to a portion of cases, thus, leading to a misdiagnosis. 

Fetal structural anomalies found in the late second trimester or even third trimester require both timely and comprehensive genetic analysis, which is much needed for accurate genetic counseling. It may not only provide a prognosis that allows parents to make better informed choices about continuing or terminating the pregnancy, where termination is legally possible in China until 28 weeks of gestation and is possible after this time under exceptional circumstances, but also may help the obstetrician determine the best obstetric management, such as the delivery mode, and help the neonatologists in optimizing neonatal care. Recently, the procedure of simultaneous detection of CNVs and SNVs/indels following normal QF-PCR results has been applied in prenatal diagnosis [19]. In our cohort of study, only 16 cases with ultrasound anomalies were found in the early gestation (≤14 weeks), while 67 fetuses were found in the second trimester and even in the third trimester. In these cases, it is necessary and feasible to perform simultaneous detection of CNVs and SNVs/indels procedure for prenatal genetic diagnosis to provide more timely and comprehensive genetic counseling to the parents.

Ultrasonic fetal soft markers are defined as a minor structural change, usually transient, which might be seen in the normal fetus. These markers were used to screen for Down syndrome and other aneuploidies. Standardized ultrasound measurement of NT thickness is used in the first trimester to calculate the risk for fetal chromosomal aneuploidy. Besides, an increased NT (≥3 mm) is also associated with microdeletion/microduplication syndromes and monogenic disorders [20,21,22]. In our cohort of study, the genetic analysis indicated the presence of VOUS CNVs and negative CES in two cases of isolated increased NT (case #24 and #28). After genetic consultation, both the two families selected continuing the pregnancy and gave birth to healthy newborns. In the 4 cases of isolated cystic hygroma in our cohort, the results of the genetic analysis were all negative. The pregnancy outcomes after genetic consultation were TOP in one case, follow up loss in one case, and two healthy newborns with resolved thickening of subcutaneous neck tissue. Therefore, results from the comprehensive CNVs and SNVs/indels analysis, either negative or positive, were informative for risk evaluation, follow-up and genetic consultation. 

Parental origin of a variant plays an important role for evaluating its pathogenesis when establishing the genotype-phenotype and casual-effect relationships. Because of the heterogeneous phenotypes and incomplete penetrance in some microdeletion/microduplication syndromes, pregnancy outcome may not be determined on the proband result alone. In our cohort of study, among the CNVs detected in 16 cases, the mode of inheritance was confirmed by further testing parental samples by CMA only in 9 of the 16 families, while the other 7 parents refused further testing. It was worth noting that CNVs in these 7 cases of unknown genetic sources were all further analyzed and confirmed by CES data from their parental blood samples. Combined with the results of exome sequencing along with the parental origins of genetic analysis provide whether mutations were de novo or inherited, which is essential for genetic consulting. This information aided in determining the recurrence risk, especially to those couples who had a history of abnormal pregnancy outcome or birth defect.

Several studies have shown that a relatively high diagnosis rate by exome sequencing in specific subcategories of fetal ultrasound anomalies, such as skeletal dysplasia [3,4,23,24]. Our study also showed this high molecular diagnosis rate in subgroups was 69.2% (9/13) in the skeletal anomaly group. This may be explained by the fact that anomalies in skeletal system are more likely to be caused by single gene variants.

### 4.2. Evaluation of CNVs by CNV-Seq vs. CMA 

CMA has been recommended as the first-line method for detecting chromosomal CNVs, and SNP-array allows for detecting most cases of UPD, loss of heterozygosity (LOH) and low-level mosaic aneuploidies. CNV-seq technology based on NGS is a newly developed method for genome-wide CNV detection, which is characterized by its higher throughput, higher resolution, and lower cost than the CMA platform. In our cohort of study, a comparison of the two methods in detecting CNVs indicates that the breakpoints of CNVs were very similar, and sizes of CNVs detected by CNV-seq was slightly larger (101.0% on average) than that of CMA platform. Therefore, CMA and CNV-seq are both effectively in detecting CNVs in prenatal samples [10]. However, CNV-seq method is not capable of detecting UPD when compared to SNP assay. Generally, DNA extracted from invasively obtained fetal samples were of relatively poor quality, and non-amplified DNA samples are required for CNV-seq analysis. Compared with CMA, CNV detection using CNV-seq may be advantageous in prenatal samples with poor DNA quality and/or limited sample size [9]. Additionally, the cost per sample of CNV-seq (USD 300–375) was less than CMA (USD 600–750). This support the replacement of CMA by low-pass genome sequencing for molecular cytogenetic testing [25].

### 4.3. Evaluation of SNVs/Indels by CES vs. WES

Exome sequencing was recommended as the method for prenatal DNA sequencing by the ACMG. WES and CES are routinely used for the genetic diagnosis of severe monogenic disorders. According to the clinical laboratory standards and guidelines developed by the ACMG, WES analysis mainly focuses on approximate 4000 genes associated with Mendelian disease in the OMIM database. In the present study, CES was a custom designed panel, which included these human disease-causative genes found in the OMIM, the Human Gene Mutation Database (HGMD) databases and new peer-reviewed literature, all genes had exonic regions and 30-bp of the exon-intron boundaries covered, but as well as the known pathogenic deep intronic variations [26,27]. This approach represents a more effective strategy to clinical implementation. A comparison between CES and WES was performed in 52 cases in this study, and the variants analyzed by WES were all in accordance with CES, which indicates that either CES or WES was appropriate method to diagnose of Mendelian disorders. It is known that CES and WES were capable of detecting CNVs based on read-depth information from sequencing data [28,29,30]. However, the average sequencing depth of CES is 200× and those of WES is 100×, and CES has been shown reliably detect CNV due to the relatively higher average sequencing depth. As a result, the genetic source of CNVs detected by CMA or CNV-seq in fetal samples were confirmed via CNVs analysis from peripheral blood samples of parents by trio-based CES.

### 4.4. Issues to Be Considered for Clinical Implementation 

The size of our current patient cohort is relatively small. While the diagnosis yield for each mutation category is relatively consistent with previous study in general, but different from that of PAGE and Columbia large cohort [3,4]. Thus, it will be necessary to apply our approach to a larger number of patients to address the clinical utility. 

Assessment of the genetic etiology of case with prenatal ultrasound findings is essential for appropriate management and counseling. While there have been advances in genetic technologies, still at its infancy, with this current study we focused on isolated cases report and a small patient cohort. There is an urgent need to have the evidence-based evaluation of the implementation of sequencing-based technology for a prenatal workup. 

The interpretation of rare VOUS variant is a major barrier in prenatal genetic diagnosis. Reported VOUS during pregnancy was expected to increase parental anxiety and hamper parental decision-making. The detection rate of VOUS associated with fetal phenotypes was 18.1% (15/83) in our cohort study. In 13 cases with VOUS results obtained from CMA, we received a molecular diagnosis in 3 cases, with a diagnostic yield of 23.1% (3/13). The clinical features of the disease associated with these 3 variants were all concordant with the fetal ultrasound findings. We currently chosen to report VOUS for the reason that the variant was likely to contribute to the fetal phenotype. Since rare VOUS were unavoidable and challenges for genetic testing, we have to face the problem and gain more experience and effort to address this obstacle.

Fetuses with positive ultrasound finding, but with negative genetic result after CNVs and SNVs/indels analysis remains a challenge in terms of calculating the residual risk and appropriate follow-up. First, our current patient cohort is still small. The diagnosis yield for each mutation category is relatively consistent with previous study in general, but different from that of previous PAGE and Columbia large cohort study. Thus, it will be necessary to apply our approach to a larger cohort for clinical evaluation.

## 5. Conclusions 

Our study demonstrated that a reasonable prenatal diagnosis with fetal ultrasound anomalies is largely depending on a relatively timely and complete genetic variation information, including the analysis of karyotype, CNV and SNV simultaneously. Conventional karyotype analysis has huge advantages for the identification of balanced chromosomal aberrations (including translocations and inversions), chromosomal mosaic types (especially sex chromosomes), and multiploid chromosomes. Therefore, karyotyping analysis could not be replaced by CMA or CNV-seq in prenatal diagnosis of fetal ultrasound abnormalities. Cases with VOUS CNVs still need further exome sequencing. The combination of CNVs and SNVs/indels may improve the diagnostic yield of normal karyotypic fetus with ultrasound anomalies, while monogenic diseases occupied the majority of cases. As a result, simultaneous detection of CNVs (using CMA or CNV-seq) and SNVs (using CES or WES) are recommended for prenatal genetic diagnosis in fetuses with ultrasound anomalies and normal karyotypes.

## Figures and Tables

**Table 1:** Classification of fetal ultrasound anomalies, molecular diagnostic rate and pregnancy outcomes.

Phenotype Category	Cases (n)	CMA Results		Trio-Based CES Results		Molecular Diagnostic Rate (%)	% of VOUS	Pregnancy Outcomes		
		P/LP	VOUS	P/LP	VOUS			TOP	Livebirth	Lost to Follow-Up
**Fetal soft markers**	**9**	**1**	**4**	**1**	**0**	**22.2% (2/9)**	**33.3% (3/9)**	**33.3% (3/9)**	**44.5% (4/9)**	**22.2% (2/9)**
Isolate increased NT (≥3 mm)	5	1	4	1	0	40.0% (2/5) *	60.0% (3/5)	2	2	1
Cystic hygroma ^#^	4	0	0	0	0	0	0	1	2	1
**Fetal structural anomalies**	**74**	**2**	**9**	**26**	**5**	**36.5% (27/74)**	**16.2% (12/74)**	**90.5% (67/74)**	**8.1% (6/74)**	**1.4% (1/74)**
Facial anomaly	4	0	2	0	0	0	50.0% (2/4)	2	1	1
Brain anomaly	13	2	0	2	2	30.8% (4/13)	16.7% (2/12)	12	1	
Skeletal anomaly	13	0	1	9	0	69.2% (9/13)	7.1% (1/14)	12	1	
Cardiac anomaly	4	0	0	1	0	25.0% (1/4)	0	4	0	
Renal anomaly	4	0	2	3	1	75% (3/4)	25.0% (1/4) **	3	1	
Hydrops	8	0	1	2	0	25.0% (2/8)	12.5% (1/8)	8	0	
Early-onset FGR	5	0	3	0	0	0	60.0% (3/5)	3	2	
Overgrowth	1	0	0	1	0	/	/	1	0	
Multi-systemic anomalies (≥2 structural anomalies)	22	0	0	8	2	36.4% (8/22)	9.1% (2/22)	22	0	
**Total**	**83**	**3**	**13**	**27**	**5**	**36.1% (30/83)**	**18.1% (15/83)**	**84.3% (70/83)**	**12.0% (10/83)**	**3.6% (3/83)**

^#^ Fetal cystic hygroma is a congenital malformation of the lymphatic system characterized by bilateral fluid-filled jugular lymphatic sacs of the fetal neck, without internal septations. P/LP, pathogenic/likely pathogenic; VOUS, variants of uncertain significance; * one case with VOUS CNV and P SNV; ** one case with VOUS CNV and LP SNV, one case with VOUS for CNV and SNV.

**Table 2:** Fetal phenotypes and pregnancy outcomes in 30 cases with P/LP variants and 15 cases with VOUS detected by CMA and CES.

ID	Phenotypic Category	Phenotypic Descriptions	Sample Types	Gestational Weeks at Testing (w)	Molecular Genetic Analysis				Pregnancy Outcomes
					Variants	Origin	Classification	Diseases; Inherited Pattern	
12	Isolated increased NT	NT = 3.5 mm	Amniotic fluid	17	arr 16p11.2(29,567,296-30,190,029)×1	De novo	P	16p11.2 deletion syndrome	Lost to follow-up
14	Isolate increased NT	NT = 3.3 mm	Amniotic fluid	17	arr 22q11.23(23,692,307-25,039,015)×3 *FLT4* [NM_182925]: c.1966C>T (p.Q656*); Mosaic (26.0%)	Maternal De novo	VOUS P	22q11.23 duplication syndrome Lymphatic malformation; AD	TOP
24	Isolate increased NT	NT = 3.2 mm	Chorionic villus	14	arr Xq26.3q27.1(137,785,138-138,629,978)×2; Male	N/A	VOUS	Xq26.3q27.1 duplication syndrome	Livebirth
27	Isolate increased NT	NT = 6.0 mm	Chorionic villus	13	arr 16q23.3q24.1(83,734,648-85,746,186)×1 arr 22q11.23(23,698,548-24,992,266)×3	N/A N/A	VOUS VOUS	16q23.3q24.1 deletion syndrome 22q11.23 duplication syndrome	TOP
28	Isolate increased NT	NT = 5.3 mm	Amniotic fluid	17	arr 15q11.2(22,770,421-23,282,798)×1	Paternal	VOUS	15q11.2 deletion syndrome	Livebirth
11	Facial anomaly	Orbital hypertelorism	Amniotic fluid	18	arr 15q21.3(57,312,840-58,070,661)×3	Maternal	VOUS	15q21.3 duplication syndrome	Livebirth
26	Facial anomaly	Cleft lip and alveolar	Amniotic fluid	26	arr 18p11.31(3,190,315-3,520,287)×3	N/A	VOUS	18p11.31 duplication syndrome	Lost to follow-up
37	Brain anomaly	Microcephaly, small cerebellum	Cord blood	25	*ASPM* [NM_018136]: c.3598+1G>T; Het c.7782_7783del (p.K2595Sfs*6); Het	Paternal Maternal	P P	Primary AR microcephaly	TOP (autopsy showed microcephaly)
62	Brain anomaly	Cerebellar hypoplasia, Dandy-Walker malformation	Amniotic fluid	18	arr 16p11.2(29,591,326-30,176,508)×3	Paternal	P	16p11.2 duplication syndrome	TOP
76	Brain anomaly	Small cerebellum, abnormal brain sulci	Amniotic fluid	27	arr 6q26q27(161,323,190-171,114,867)×1	De novo	P	6q26q27 deletion syndrome	TOP
80	Brain anomaly	Agenesis of the corpus callosum	Amniotic fluid	24	*EPG5* [NM_020964]: c.2461C>T(p.R821*); Het c.88C>T(p.Q30*); Het	Paternal Maternal	P P	Vici syndrome; AR	TOP
78	Brain anomaly	Spinal bifida	Amniotic fluid	18	*PTCH1* [NM_000264]: c.763C>T (p.R255W); Het	Maternal	VOUS	Holoprosencephaly 7; AD	TOP
83	Brain anomaly	Subependymal nodules in the left ventricle, mild left ventriculomegaly	Cord blood	30	*RLIM* [NM_016120]: c.1864A>G (p.S622G); Het	De novo	VOUS	Tonne-Kalscheuer syndrome; AD	Livebirth
3	Skeletal anomaly	Skeletal abnormality of lower extremity	Chorionic villus	12	*SOX9* [NM_000346]: c.431+1G>T; Het	De novo	LP	Campomelic dysplasia with autosomal sex reversal; AD	TOP
4	Skeletal anomaly	Abnormal morphology of skull and rib, small thoracic cage, short and curved long bones	Amniotic fluid	20	*FGFR3* [NM_001163213]: c.746C>G (p.S249C); Het	De novo	P	Thanatophoric dysplasia; AD	TOP
18	Skeletal anomaly	Short long bones	Cord blood	30	*FGFR3* [NM_001163213]: c.1144G>A (p.G382R); Het	De novo	LP	Hypochondroplasia; AD	TOP
20	Skeletal anomaly	NT = 3.1 mm, scalp edema, abnormally flexed limbs	Chorionic villus	13	*COL1A1* [NM_000088]: c.3541G>A (p.G1181S); Het	De novo	LP	Osteogenesis imperfecta; AD	TOP
41	Skeletal anomaly	Significantly shorter ulna than the tibia	Amniotic fluid	20	*ALPL* [NM_000478]: c.346G>A (p.A116T); Het c.476A>T (p.K159I); Het	Maternal De novo	P LP	Infantile hypophosphatasia; AR	TOP (autopsy showed a wide eye distance, small jaw, prominent forehead, bent forearm, rocker bottom feet, shorter ulna than the tibia)
64	Skeletal anomaly	Short long bones, hydramnios	Amniotic fluid	23	*FGFR3* [NM_000142]: c.1138G>A (p.G380R); Het	De novo	P	Achondroplasia; AD	TOP
66	Skeletal anomaly	Short limbs	Amniotic fluid	27	*FGFR3* [NM_001163213]: c.742C>T (p.R248C); Het	De novo	P	Achondroplasia; AD	TOP
67	Skeletal anomaly	Abnormal morphology of skull	Cord blood	26	*FGFR2* [NM_000141]: c.1025G>T (p.C342F); Het	De novo	P	Craniosynostosis; AD	TOP
70	Skeletal anomaly	Short and curved femur	Cord blood	28	*COL1A1* [NM_000088]: c.633delT (p.G211Efs*53); Het	Paternal	LP	Osteogenesis imperfecta; AD	TOP
23	Skeletal anomaly	Talipes varus of right foot	Amniotic fluid	26	arr 7p21.3(9,245,562-10,638,811)×3	N/A	VOUS	7p21.3 duplication syndrome	Livebirth
77	Cardiac anomaly	Cardiac rhabdomyoma	Amniotic fluid	22	*TSC2* [NM_000548]: c.4318C>T (p.Q1440*); Het	De novo	P	Tuberous sclerosis; AD	TOP
15	Renal anomaly	Left polycystic kidney	Cord blood	25	arr 8p22(17,910,003-18,535,000)×3 *DSTYK* [NM_015375]: c.1324+2T>C; Het	Maternal Maternal	VOUS LP	8p22 duplication syndrome Congenital anomalies of kidney and urinary tract 1; AD	TOP
33	Renal anomaly	Bilateral renal enlarged, cystic changes, oligohydramnios	Cord blood	23	*PKHD1* [NM_138694] : c.4437_4440del (p.F1479Lfs*20); Het c.5935G>A (p.G1979R); Het	Maternal Paternal	LP LP	Polycystic kidney disease 4, with or without hepatic disease; AR	TOP (autopsy showed polycystic kidney)
51	Renal anomaly	Bilateral renal enlarged, echogenicity increased, unclear boundary between cortex and medulla	Cord blood	33	*PLCE1* [NM_016341]: c.3019A>G (p.R1007G); Het c.4037_4039del (p.1346_1347del); Het	Maternal Paternal	VOUS LP	Nephrotic syndrome; AR	TOP
25	Renal anomaly	Polycystic right kidney	Cord blood	25	arr 8p21.2(23,899,930-25,114,973)×3 *LRP5* [NM_002335]: c.3514C>T (p.R1172C); Het	N/A Paternal	VOUS VOUS	8p21.2 duplication syndrome Polycystic liver disease 4 with or without kidney cysts; AD	Livebirth
34	Hydrops	Subcutaneous edema, hydrothorax	Amniotic fluid	24	*RAPSN*[NM_005055]: c.280G>A (p.E94K); Het c.288delG (p.C97Afs*31); Het	Maternal Paternal	LP LP	Fetal akinesia deformation sequence/Congenital myasthenic syndrome; AR	TOP
44	Hydrops	Subcutaneous edema in the head and trunk, hydrothorax	Amniotic fluid	19	*PIEZO1* [NM_001142864]: c.145C>T (p.R49*); Het c.91_92delTC (p.S31Afs*94); Het	Paternal Maternal	LP LP	Lymphatic malformation; AR	TOP
22	Hydrops	NT = 6.0mm, subcutaneous edema all over the body	Chorionic villus	12	arr 4p14p13(39,328,869-43,416,810)×4	N/A	VOUS	4p14p13 duplication syndrome	TOP (thickness of subcutaneous tissue of neck)
13	Early-onset FGR	Fetal growth restriction	Cord blood	30	arr Xq26.2(130,586,055-131,231,800)×2; Male	Maternal	VOUS	Xq26.2 duplication syndrome	Livebirth
16	Early-onset FGR	Fetal growth restriction	Cord blood	30	arr 3p21.1(53,645,774-54,264,966)×3	Maternal	VOUS	3p21.1 duplication syndrome	Livebirth
54	Early-onset FGR	Fetal growth restriction	Cord blood	26	arr Xp22.13p22.12(18,646,432-19,407,076)×3; Female	Maternal	VOUS	Xp22.13p22.12 duplication syndrome	TOP
9	Overgrowth	Hydramnios, overgrowth	Cord blood	30	*HRAS* [NM_001130442]: c.35G>T (p.G12V); Het	De novo	P	Costello syndrome; AD	TOP
2	Multi-systemic anomalies	Ventriculomegaly, cerebellar medulla pool widened, separated cerebellar hemisphere, Ventricular septal defect, renal pelvis broadening	Chorionic villus	12	*SETD2* [NM_001349370]: c.5086C>T (p.R1696W); Het	De novo	LP	Luscan-Lumish syndrome; AD	TOP
7	Multi-systemic anomalies	NT = 4.5 mm, small inner diameter of the eyes, irregular morphology of vitreous body, mild hydropericardium	Amniotic fluid	19	*CHD7* [NM_017780]: c.2753_2756delinsTGG (p.W918Lfs*7); Het	De novo	LP	CHARGE syndrome; AD	TOP
8	Multi-systemic anomalies	Meningocele, irregular morphology of occipital skull	Chorionic villus	13	*RPGRIP1L* [NM_015272]: c.1421dupA (p.N474Kfs*12); Het c.1421delA (p.N474Mfs*7); Het	Maternal Paternal	LP LP	Meckel syndrome; AR	TOP (autopsy showed cleft palate, polydactyly, absence of nasal bridge, renal cysts; clincial diagnosis of Meckel syndrome)
35	Multi-systemic anomalies	NT = 3.7 mm, hydrops, abnormal wrist joints and bipedal posture	Chorionic villus	12	*CHRNA1* [NM_001039523]: c.119G>A (p.R40Q); Homo	Paternal and maternal	LP	Multiple pterygium syndrome/Congenital myasthenic syndrome; AR	TOP
36	Multi-systemic anomalies	Dandy-Walker malformation, four fingers on the left hand, hydropericardium	Cord blood	27	*NIPBL* [NM_133433]: c.4051C>T (p.Q1351*); Het	De novo	LP	Cornelia de Lange syndrome; AD	TOP
40	Multi-systemic anomalies	Cardiac rhabdomyoma, multiple nodules in bilateral paraventricular and bilateral prefrontal cortex	Amniotic fluid	23	*TSC2* [NM_000548]: c.4662+1G>A; Het	De novo	P	Tuberous sclerosis; AD	TOP
53	Multi-systemic anomalies	NT = 6.0 mm, subcutaneous edema all over the body, abnormal spinal alignment	Chorionic villus	14	*BMP2* [NM_001200]: c.313C>T (p.R105*); Het	De novo	LP	Short stature, facial dysmorphism, and skeletal anomalies; AD	TOP
56	Multi-systemic anomalies	Micrognathia, single umbilical artery, irregularly arranged sacrococcygeal vertebral bodies, increased renal cortical echogenicity	Cord blood	25	*KMT2D* [NM_003482]: c.2317dupC (p.Q773Pfs*3); Het	De novo	LP	Kabuki syndrome; AD	TOP
65	Multi-systemic anomalies	Short humerus and femur, narrow chest, diffuse enhancement of echo in both kidneys, bilateral pulmonary dysplasia, left ventricular dysplasia	Amniotic fluid	24	*DYNC2H1* [NM_001080463]: c.4625C>T (p.A1542V); Het c.10894G>C(p.A3632P); Het	Maternal Paternal	VOUS VOUS	short-rib thoracic dysplasia 3 with or without polydactyly; AR	TOP
79	Multi-systemic anomalies	Occipital meningocele, bilateral polycystic kidney, multiple fingers/toes, oligohydramnios	Amniotic fluid	20	*TMEM67* [NM_001142301]: c.932C>G (p.P311R); Het c.2083T>C (p.S695P); Het	Maternal Paternal	VOUS VOUS	Meckel syndrome; AR	TOP

* Termination codon; P/LP, pathogenic/likely pathogenic; VOUS, variants of uncertain significance; AR, autosomal recessive; AD, autosomal dominant; Het, heterozygous; Homo, homozygous; TOP, termination of pregnancy; N/A (Not applicable).

**Table 3:** Comparison of breakpoints and sizes of CNVs detected by two platforms of CMA and CNV-seq.

Case ID	Phenotype Category	Sample Types	Gestational Weeks at Testing (w)	CNVs	CMA Results		CNV-Seq Results	
					CNV Breakpoints	Size (kb)	CNV Breakpoints	Size (kb)
11	Facial anomaly	Amniotic fluid	18	15q21.3 Dup (×3)	57,312,840–58,070,661	757	57,325,003–58,075,000	749
13	Early-onset FGR	Cord blood	30	Xq26.2 Dup (×2, male)	130,586,055–131,231,800	645	130,560,003–131,235,000	674
15	Renal anomaly	Cord blood	25	8p22 Dup (×3)	17,910,003–18,535,000	624	17,906,398–18,529,463	624
16	Early-onset FGR	Cord blood	30	3p21.1 Dup (×3)	53,645,774–54,264,966	619	53,660,000–54,285,000	625
22	Isolated increased NT (≥3mm)	Chorionic villus	12	4p14p13 Dup (×4)	39,328,869–43,416,810	4087	39,307,003–43,432,700	4125
23	Skeletal anomaly	Cord blood	26	7p21.3 Dup (×3)	9,245,562–10,638,811	1393	9,285,003–10,685,000	1399
24	Isolated increased NT (≥3 mm)	Chorionic villus	14	Xq26.3q27.1 Dup (×2, male)	137,785,138–138,629,978	844	137,785,003–138,635,000	849
25	Renal anomaly	Cord blood	25	8p21.2 Dup (×3)	23,899,930–25,114,973	1215	23,910,003–25,110,000	1199
27	Isolated increased NT (≥3 mm)	Chorionic villus	13	16q23.3q24.1 Del (×1)	83,734,648–85,746,186	2011	83,735,003–85,760,000	2024
				22q11.23 Dup (×3)	23,698,548–24,992,266	1293	23,775,003–24,950,000	1176
54	Early-onset FGR	Cord blood	26	Xp22.13p22.12 Dup (×3, female)	18,646,432–19,407,076	760	18,619,530–19,395,529	775
62	Brain anomaly	Amniotic fluid	19	16p11.2 Dup (×3)	29,591,326–30,176,508	585	29,522,292–30,199,554	677
76	Brain anomaly	Amniotic fluid	27	6q26q27 Del (×1)	161,323,190–171,114,867	9791	161,323,355–171,115,067	9791

Dup, duplication; Del, deletion.
